# Propolis alleviates brain tissue damage and oxidative abnormalities in streptozotocin (STZ)-induced diabetes

**DOI:** 10.1016/j.jgeb.2026.100663

**Published:** 2026-02-09

**Authors:** Ahmed M. Ashour

**Affiliations:** Department of Pharmacology and Toxicology, College of Pharmacy, Umm Al-Qura University, Makkah, Saudi Arabia

**Keywords:** Diabetes, Brain damage, Streptozotocin, Propolis, Ischemia

## Abstract

**Background:**

Diabetes is a serious and rapidly growing global health issue that can affect many organs, including the brain. It is often linked to complications such as cardiovascular disease and cerebral ischemia. Propolis, a natural resin produced by honey bees, is rich in phenols and flavonoids known for their antioxidant, anti-inflammatory, and immune-modulating properties.

**Objective:**

This primary aim in this research was to evaluate the neuroprotective effects of propolis on brain tissue in rats that have diabetes which is introduced through nicotinamide (NA) and streptozotocin (STZ).

**Methods:**

Male Wistar rats were separated into categories. Except for the control category, all were fed a diet that had high fat levels. Diabetes was induced using intraperitoneal injections of NA and STZ. After induction, diabetic rats received oral propolis at doses of 50 or 100 mg/kg daily for eight weeks. Throughout the study, lipid profiles, fasting blood glucose, insulin levels, and oxidative stress markers were assessed by taking measurements. At the end of the experiment, brain tissues were analyzed for cytokine levels, antioxidant activity, and DNA damage using the COMET assay, in addition to histopathological and immunohistochemical examinations.

**Results:**

Treatment with propolis significantly reduced fasting blood glucose and insulin levels, improved profiles of lipid, and decreased oxidative stress and inflammatory mediators. Histological analysis showed that rats treated with propolis had noticeably less brain tissue damage compared to untreated diabetic rats.

**Conclusion:**

Propolis demonstrated clear neuroprotective effects in diabetic rats, likely through its antioxidant and anti-inflammatory mechanisms. These findings suggest that propolis may present a considerably potential solution as a natural therapeutic agent for reducing diabetes-induced brain damage.

## 1 Introduction

Across public health, diabetes is among the most critical metabolic ailments since it is becoming more and more widespread globally. Diabetes raises the danger relating to cerebral ischemia brought on by either cardiovascular disorders (CVD) or ischemic stroke. The involvement of various toxic pathways, such as stress from oxidative processes, impairment of the leukocyte function, angiogenesis abnormal nature, a rise in blood–brain barrier (BBB) permeability, and inflammatory response, it has also been reported that hypoglycemia and diabetes can worsen the damage that has already occurred as a result of cerebrovascular disorder.^1^, ^2^

Diabetes secondary complications are brought on by long-term diabetes. Heart disease, a higher likelihood of stroke, blindness, hypoglycemia, amputations, renal failure, and troubles with the central nervous system (CNS) are just a few of the health problems that can result from this illness. Structure-related changes or brain shrinkage, as well as electrophysiological property changes, may be evidence of diabetes-related CNS problems; these deficits in cognitive function are the end outcome.^3^

Multiple factors have been linked to the type 2 diabetes’ (T2DM) pathogenesis. These include a higher body mass index (BMI), a lack of exercise, cigarette smoking a high-glucose and high-fat diet, genetic vulnerability, old age, and drug side effects. Nevertheless, consumption of high-glucose/high-fat diets and obesity, are the most prevalent risk factors. This may cause the pancreatic beta cells to produce insulin continuously and desensitize the insulin receptors as a result. Overstimulation of β-cells, the cells involved with the production insulin, causes cellular death overtime, which can worsen the condition.^4^, ^5^

Major comorbidities, including diabetes, for stroke, are related to increased proinflammatory cytokines notably tumor necrosis factor (TNF-α), interferon (IFN), interleukin-1 (IL-1), and interleukin-6 (IL-6), alongside shifted activation of the macrophages and other immune cell populations.^6^

Propolis, a sticky resinous material, is manufactured by the honey bees from both the leaves and flowers’ resin in trees after being mixed with their saliva, to smooth out walls, plug cracks, and maintain humidity and temperature balance inside the hives.^7^

For many years, propolis has found use in traditional medications around the globe. Propolis comes in a wide variety of chemical compositions from different geographical locations and seasons, providing a wide range of options when picking the appropriate type for a given application. Owing to its abundance in specific components, notably flavonoids and phenols, which have antioxidant, anti-inflammatory, immunomodulatory, antiproliferative, antiseptic, antimicrobial, and wound-healing properties, propolis is a better option for treating a range of conditions.^8^

Propolis contains more than 300 substances that are potentially active, notable ones being, phenolic aldehydes, amino acids, coumarins, steroids, and polyphenols. Propolis has been used for many years, for a variety of applications from being utilized as a booster for immune to use as an anti-inflammatory, antibacterial, anti-cancer, as well as anti-oxidant, essentially due to its possible therapeutic capabilities.^9^ In order to experimentally replicate the metabolic and pathophysiological features of type 2 diabetes mellitus, the nicotinamide-streptozotocin (NA–STZ) model is widely employed in rodents. In this model, nicotinamide is administered prior to streptozotocin to partially protect pancreatic β-cells from complete destruction, accordingly helping induce moderate hyperglycemia, residual insulin secretion, and insulin resistance that closely resemble human T2DM rather than type 1 diabetes.^10^, ^11^ To reduce acute hypoglycemic mortality following STZ administration, animals are routinely provided with glucose supplementation (5% glucose solution) for 24 h post-injection, a standard procedure that enhances survival and model reproducibility.^11^

The primary objective of this experimental study was to assess the potential neuroprotective effects of propolis against NA-STZ diabetes that is induced in rats and to examine whether propolis could reduce cerebral damage associated with diabetes by suppressing the oxidative stress and inflammatory mediators.

## 2 Methods and materials

### 2.1 Chemicals

The researchers acquired Streptozotocin from “Sigma-Aldrich, USA”. The supplier of the propolis was “Puritan's Pride, USA”. The best available analytical grade was chosen for all other compounds used in the investigation.

### 2.2 Animals

A sum of 32 male Wistar rats that were mature and having weights of between 180–200 g were bought from the house colony for animals at Umm Al-Qura University in Makkah, Saudi Arabia. The researchers kept the rats in conventional and clean housing units, at a persistent environmental temperature, with regular dark and light cycles, and free of pathogens. Food and water were made available to animals without restriction. Rats were allowed a week to become acclimated to the new habitat before the experiment began.

### 2.3 Induction of diabetes

According to Abdel-Rahman et al. (2020b, two successive intraperitoneal injections where the chemical used were nicotinamide (NA) and streptozotocin (STZ), were used to induce diabetes in overnight-fasted animals. NA had been dissolved in sterile saline. A treatment solution of STZ (45 mg/kg) in 0.1 M-citrate buffer (pH 4.5), which was freshly prepared, was administered to rats 15 min intraperitoneally before NA (110 mg/kg) was administered.^12^, ^13^ Except for the animals in the control group, where the animals received only the vehicle (in this case the water which was distilled), all animals received injections of NA-STZ. Next a 6-hour NA-STZ injection, rats were given unrestricted accessibility and reach to a 10-percent (w/v) solution of glucose for the following 24-hour period. The level of fasting blood glucose (FBG) was assessed in accordance to Trinder after 48 h of NA-STZ injection.^53^ Rats with FBG levels of more than 200 mg/dL were designated as having successfully been induced with diabetes for experimental purposes, and were allocated further screening and experiments.^62^

The rats successfully induced with diabetes were given a high-fat diet (HFD) that was consisted 41% fat, 41% carbohydrate, as well as 18% protein (percentages here defined in terms of all kcal of the HFD). Negative control rats received standard diet containing 3% fat and a total of 48.8% carbohydrates, as well as 21% proportion of proteins.

### 2.4 The study groups

The rats successfully induced with diabetes and provided with the above food materials were allocated into four groups, of eight rats each in a random manner. The first two categories, which were assigned as the negative and diabetic control groups (NC and STZ/NA-DC), respectively, were orally provided with the vehicle for the course of the 8-week experimentation. Respectively, propolis was given orally to rats in groups 3 and 4 in dosages of 50 and 100 mg/kg per day. Four and eight weeks following the initiation of the drug, FBG, and the levels of insulin were assessed.

### 2.5 Collection of blood samples and determination of FBG, insulin and HOMA-IR

After eight weeks, blood was taken from the tail vein under mild anesthesia into sampling tubes, and sera were separated for the determination of insulin level by centrifuging at 3500 rpm for 15 min,^7^ and based on the following equation, HOMA-IR was calculated: [fasting glucose (mg/dL) × 0.0555 × fasting serum insulin (mUI/L)/22.5] according to Matthews et al. ^16^.

### 2.6 Euthanasia and samples of brain tissue

Anesthetized animals were decapitated after blood was drawn, and their brain hemispheres were immediately dissected on ice plates.^43^ Rat brains were utilized for the analysis of the cytokines (BDNF, TNF-α, and NSE) and antioxidant markers (MDA, GSH, and SOD) as well as other parts of the brain were utilized for the evaluation of histopathology, and immunohistochemistry.

### 2.7 Determination of lipid profiles cholesterol, triglycerides, HDL and LDL

Serum lipid profile; low density lipoproteins (LDL), lipoproteins (HDL) of high density, cholesterol and triglycerides (TGs); were assessed colorimetrically through the use of kits (Reactivos-GPL, Barcelona-Spain).

### 2.8 Preparation of brain tissue homogenates

The preparation of the brain tissue homogenate was done under the guidelines of the tool defined by Mansour et al. ^18^. Brain tissues from 6 animals per group were assembled and lavaged with ice-cold saline for the biochemical analyses. A 10% (w/v) brain tissue homogenate was produced in 0.1 M phosphate buffer (pH 7.4). A cooling centrifuge (2 k15; Sigma/Laborzentrifugen) was employed for centrifuging the homogenates for a period of 15 min at 10,000 rpm. The researchers separated and kept the resultant supernatants at −80 °C up to the time they could be examined further. The content of protein in the brain tissue homogenate was quantified through the use of the Bradford assay. The resulting brain extract supernatant was stored at −80 °C until analysis.^12^

### 2.9 Determination of brain oxidative enzymes and lipid peroxidation contents

In accordance with^48^ study,^48^ the activities of superoxide dismutase (SOD) were assessed in brain homogenate. The Ellman method^17^ was used to measure the reduced glutathione (GSH) level. The amount of malondialdehyde (MDA) in the brain tissue was used to measure the lipid peroxidation products.^28^

### 2.10 Determination of neuron specific enolase (NSE) activity in serum and brain

Through the use of Enzyme-Linked Immunosorbent Assay (ELISA) kits commercially obtained, Neuron-specific enolase (NSE) was measured in serum samples and brain homogenate (Biogen Co., Egypt); all kit instructions were followed.

### 2.11 Determination of C-reactive protein level (CRP) in serum and brain

Following the manufacturer's instructions, ELISA kits that are tailored for rat CRP (Biogen Co., Egypt) and commercially available were used to measure the levels of CRP in serum and brain tissue. A standard curve created using the kit's known CRP standards was used to establish the quantity of CRP present in serum specimens. To account for variances in tissue handling and extraction effectiveness, CRP levels in brain tissue were standardized to tissue protein concentration.

### 2.12 Determination of homocysteine level in serum and brain

Using a commercially available homocysteine-specific enzymatic assay kit (Sigma-Aldrich, Catalog No. MAK367), the levels of homocysteine in serum and brain homogenate were determined in accordance with the manufacturer's guidelines for reagent and standard preparation. A standard curve created from known homocysteine standard concentrations was used to calculate homocysteine concentrations.

### 2.13 Determination of brain-derived neurotropic (BDNF) factor and tumor necrosis factor alpha (TNF-α) in brain

Through the use of commercially available Elabscience Biotechnology Co., Ltd., USA’s ELISA kits, the BDNF and TNF-α were assessed in brain homogenates. Manufacture instructions were adhered to during this assessment.

### 2.14 Histopathological examination of brain tissue

The brain tissue of two rats from each category was preserved for 24 h in 10% neutral-buffered formalin before being stained for light microscopy. The samples were dehydrated using serial alcohol dilutions, washed with xylene, and then embedded in paraffin wax in a hot air oven set at 56 °C for six hours. A microtome was used to cut paraffin wax tissue blocks into slices of about 4–6 μ. On glass slides, the sections were subsequently put together and deparaffinized. Through the use of eosin and hematoxylin stain, they were stained for a repeat and histological inspection.^29^

### 2.15 Immunohistochemical analysis of glial fibrillary acidic protein (GFAP) in brain tissue

On positively charged slides by the avidinbiotin–peroxidase complex (ABC) technique, paraffin sections were mounted.^43^ Sections from each group were treated with mouse monoclonal anti-GFAP antibody (Servicebio, Cat# GB12100, 1:800), followed by the chemicals used in the ABC procedure (Vectastain ABC-HRP kit, Vector labs). To identify the complexes resulting in the antigen–antibody factors, the researchers used marker expression, tagging with peroxidase and coloruring with diaminobenzidine (DAB) from Sigma. The NCs were included through t non-immune serum substitution for the primary or secondary antibodies. IHC-stained slices were evaluated through the use of Olympus microscope (BX-53).

Calculations of the percentage of reaction area in ten microscopic areas using USA’s software image J 1.53 t by Wayne Rasband and National Institutes of Health for the scoring of the Immunohistochemistry data.

### 2.16 DNA fragmentation (COMET)

The Comet assay, was employed to measure DNA fragmentation in rat brain samples as previously described.^8^ Comet length (µm), head diameter (µm), tail length (µm), and tail moment were quantified to assess the extent of DNA damage, using image analysis software.

### 2.17 Statistical analysis

The researchers used the mean ± SEM approach to display the data. Graph Prism® was used for statistical analysis, which involved statistical ANOVA and Tukey's test to ascertain intergroup variability. The level of significance for the statistical measurements was set at a probability level of less than 0.05.

### 2.18 Ethical review and approval

All experimental procedures involving animals were reviewed and approved by Umm Al-Qura University the Animal Ethics Committee Approval No. HAPO-02-K-012-2023-10-1797, and were conducted in accordance with the National Institutes of Health Guide for the Care and Use of Laboratory Animals. All steps in completing the study thus aligned with the rules of Standing Committee of Bioethics Research at Umm Al-Qura University, which conforms to the National Regulations on Animal Welfare.

## 3 Results

### 3.1 Effect on blood glucose, insulin, and HOMA-IR levels

Table 1, Table 2 shows the FBG levels, and also the HOMA-IR and insulin of the experimental animals used in this study at periods of 4 and 8 weeks of the medication. Propolis significantly declined the levels of FBG relative to the STZ/NA-DC group by 73.7% and 74.2%, respectively after 4 weeks of treatment, and decreased FBG level by 74.7% and 72.2%, respectively after 8 weeks of treatment. The levels of insulin in the experimental rats induced with propolis both at the 50 mg/kg and also the 100 mg/kg were significantly increased as compared to STZ/NA-DC rats (p ≤ 0.05), by 35.7% and 71.4%, respectively after 4 weeks of treatment, and by 61.1% and 72.2%, respectively after 8 weeks. Further, HOMA-IR stabilized in a significant way in rats that were induced when evaluated in comparison to STZ/NA-DC group by 37.5% after 4 weeks of propolis administration at the two examined dose levels, and decreased by 72.0% and 56.0% after 8 weeks of treatment with propolis (50 and 100 mg/kg) respectively. Important to note, in Table 1 and other Tables and figures where we report data from the analysis, values are expressed as Mean ± SEM of six animals in each group.

**Table 1:** Effect of propolis treatment for 4 weeks on blood glucose, insulin, and HOMA-IR levels in diabetic rats.

Group	After 4 weeks		
	Blood glucose (mmol/L)	Insulin (uIU/ml)	HOMA-IR
NC	4.5^b^ ± 0.07	4.3^b^ ± 00.12	0.9^b^ ± 00.04
STZ/NA-DC	19.0^a^ ± 00.04	1.4^a^ ± 00.16	0.8^a^ ± 00.03
P-50	5.0^b^ ± 00.51	1.9^a^ ± 00.16	0.5^a^^,^^b^ ± 00.07
P-100	4.9^b^ ± 00.45	2.4^a^^,^^b^ ± 00.17	0.5^a^^,^^b^ ± 00.03

Values are expressed as Mean ± SEM of six animals in each group.

a Significantly different from the values of the negative control rats at *p* ≤ 0.05.

b Significantly different from the values of STZ/NA-DC rats at *p* ≤ 0.05.

**Table 2:** Effect of propolis treatment for 8 weeks on blood glucose, insulin, and HOMA-IR levels in diabetic rats

Group	After 8 weeks		
	Blood glucose (mmol/L)	Insulin (uIU/ml)	HOMA-IR
NC	4.7^b^ ± 00.06	4.7^a^^,^^b^ ± 00.10	1.0^b^ ± 00.03
STZ/NA-DC	27.7^a^ ± 00.58	1.8^a^ ± 00.16	2.5^a^ ± 00.07
P-50	7.0^a^^,^^b^ ± 00.49	2.9^a^^,^^b^ ± 00.30	0.7^b^ ± 00.07
P-100	7.7^a^^,^^b^ ± 00.63	3.1^a^^,^^b^ ± 00.12	1.1^b^ ± 00.17

Values are expressed as Mean ± SEM of six animals in each group.

a Significantly different from the values of the negative control rats at *p* ≤ 0.05.

b Significantly different from the values of STZ/NA-DC rats at *p* ≤ 0.05.

### 3.2 Effect on lipid profile

As shown in Table 3, Table 4, the concentrations of blood lipids (cholesterol, TGs, HDL and LDL) were raised in STZ/NA-DC rats as compared to the NC rats. On oral administration of propolis to diabetic rats, resulted in significant, statistically (*p* ≤ 0.05), improvement in serum lipids as relative to diabetic rats. Serum cholesterol and TGs, were significantly reduced to (0.8, 0.5) and (0.7, 0.4) folds in propolis treated rats (P-50 and P-100, respectively) for 4 weeks, and were significantly reduced to (0.7, 0.8) and (0.6, 0.7) folds in propolis treated rats (P-50 and P-100, respectively) for 8 weeks, as compared to DC rats.

**Table 3:** Effect of propolis treatment for 4 weeks on lipid profile in diabetic rats.

Group	After 4 weeks			
	Cholesterol (mg/dL)	TGs (mg/dL)	HDL (mg/dL)	LDL (mg/dl)
NC	85.0^b^ ± 02.41	82.4^b^ ± 00.29	72.8^b^ ± 01.70	7.6^b^ ± 00.06
STZ/NA-DC	118.2^a^ ± 05.17	109.9^a^ ± 07.22	41.5^a^ ± 02.61	18.7^a^ ± 01.71
P-50	92.1^b^ ± 05.66	59.2^a^^,^^b^ ± 04.34	54.8^a^^,^^b^ ± 02.74	16.8^a^ ± 033.40
P-100	83.7^b^ ± 06.25	49.0^a^^,^^b^ ± 04.83	51.7^a^^,^^b^ ± 02.83	16.1^a^ ± 00.94

Values are expressed as Mean ± SEM of six animals in each group.

a Significantly different from the values of the negative control rats at *p* ≤ 0.05.

b Significantly different from the values of STZ/NA-DC rats at *p* ≤ 0.05.

**Table 4:** Effect of propolis treatment for 8 weeks on lipid profile in diabetic rats.

Group	After 8 weeks			
	Cholesterol (mg/dL)	TGs (mg/dL)	HDL (mg/dL)	LDL (mg/dl)
NC	75.2^b^ ± 02.97	77.0^b^ ± 02.08	73.9^b^ ± 02.14	8.0^b^ ± 00.16
STZ/NA-DC	108.0^a^ ± 07.59	102.5^a^ ± 07.97	37.8^a^ ± 02.79	16.5^a^ ± 01.75
P-50	72.0^b^ ± 02.80	85.1^a^^,^^b^ ± 03.64	46.6^a^^,^^b^ ± 01.86	11.0^b^ ± 00.78
P-100	66.6^b^ ± 06.31	73.6^b^ ± 06.52	41.7^a^^,^^b^ ± 05.79	8.9^b^ ± 01.01

Values are expressed as Mean ± SEM of six animals in each group.

a Significantly different from the values of the negative control rats at *p* ≤ 0.05.

b Significantly different from the values of STZ/NA-DC rats at *p* ≤ 0.05.

### 3.3 Effect of propolis treatment for 8 weeks on tissue oxidative markers in diabetic rats

**Fig. 1:**
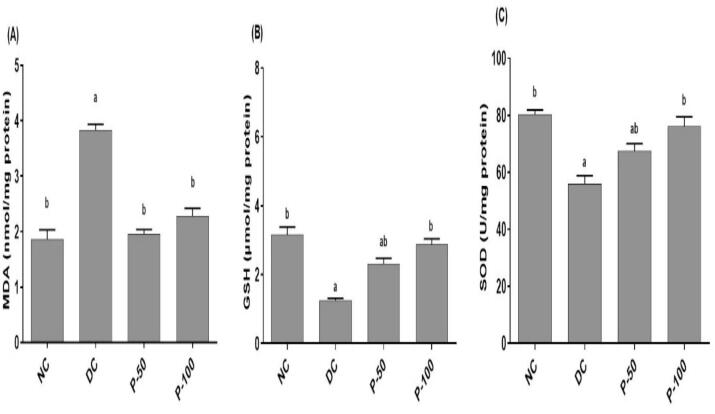
Effect of propolis on brain content of MDA GSH and SOD activity after 8 weeks in diabetic rats. ^a^Different in a significant way from the values of the negative control rats at *p* ≤ 0.05. ^b^Different in a significant way from the values of STZ/NA-DC rats at *p* ≤ 0.05.

As shown in Fig. 1, propolis (50 and 100 mg/kg) administration to diabetic rats revealed significant decrease in brain MDA levels by 39.5 and 47.3%, respectively as compared to DC rats. While, brain levels of reduced glutathione were significantly increased in propolis treated groups (50 and 100 mg/kg) in comparison to the DC group by 76.9 and 123.1%, respectively. Moreover, brain SOD activity increased in a significant way in propolis rat that had been treated with (50 and 100 mg/kg) treated groups as compared with DC group by 20.9 and 36.6%, respectively.

### 3.4 Effect of propolis treatment for 8 weeks on serum and tissue specific neuron enolase in diabetic rats

**Fig. 2:**
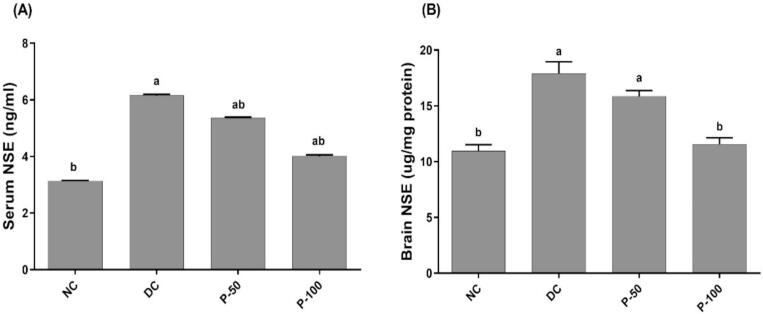
Specific Neuron Enolase (NSE) level in serum and brain tissue of control and diabetic rats treated with propolis after 8 weeks. ^a^Different in a significant way from the values of the negative control rats at *p* ≤ 0.05. ^b^Different in a significant way from the values of STZ/NA-DC rats at *p* ≤ 0.05.

As depicted in Fig. 2, both serum and brain NSE (6.2 ± 0.04 and 17.9 ± 1.05, respectively) were increased in a statistically significant way in the experiment groups rats in comparison to negative control rats (3.1 ± 0.01and 10.9 ± 0.57, respectively). On propolis (50 and 100 mg/kg) administration to diabetic rats, significant decrease in NSE serum levels by 12.9 and 35.5%, respectively was recorded as compared to DC rats. Moreover, significant (*p* ≤ 0.05) decrease in brain NSE levels by 11.2 and 35.2%, respectively was recorded in propolis (50 and 100 mg/kg) treated groups, compared to DC rats.

### 3.5 Effect of propolis treatment for 8 weeks on serum and tissue C-reactive protein in diabetic rats

**Fig. 3:**
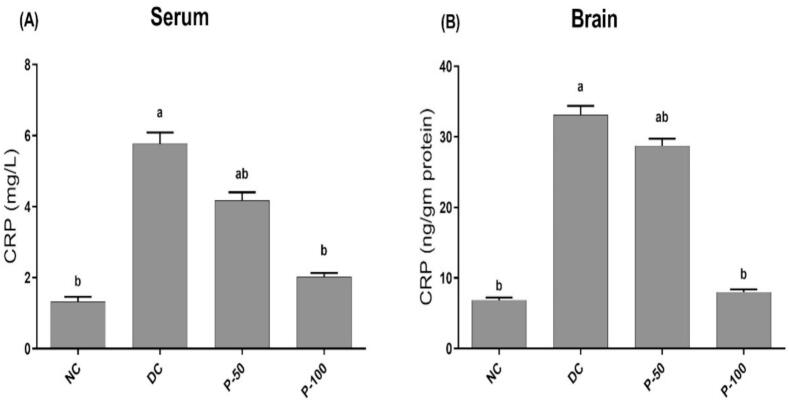
Effect of propolis treatment for 8 weeks on serum and brain tissue level of control and diabetic rats. ^a^Different in a significant way from the values of the negative control rats at *p* ≤ 0.05. ^b^Different in a significant way from the values of STZ/NA-DC rats at *p* ≤ 0.05.

Serum and brain tissue C-reactive protein (CRP) content were significantly elevated in STZ/NA-DC rats as compare to negative control rats (Fig. 3). Compared to diabetic rats, propolis administration to diabetic rats (50 and 100 mg/kg), significantly decreased serum CRP by 27.5 and 65.5%, respectively. Brain CRP contents were significantly decreased by 13.5 and 75.9%, respectively, on propolis treatment rats (50 and 100 mg/kg).

### 3.6 Effect of propolis treatment for 8 weeks on serum and tissue homocysteine in diabetic rats

**Fig. 4:**
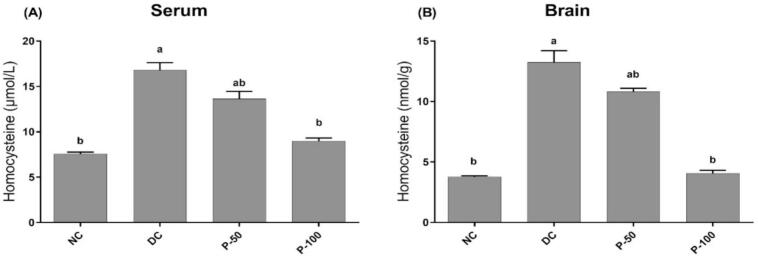
Effect of propolis treatment for 8 weeks on serum and brain tissue homocysteine level of control and diabetic rats.

Fig. 4 shows that blood and brain tissue homocysteine levels went up in diabetic rats in a significant way, statistically, relative to negative control rats. Propolis treatment to diabetic rats (50 and 100 mg/kg) significantly reduced blood homocysteine by 18.5 and 46.4%, respectively, compared to non-diabetic rats. Propolis treatment rats (50 and 100 mg/kg) revealed significantly lower brain homocysteine levels by 18.2 and 68.9%, respectively.

### 3.7 Effect of propolis treatment for 8 weeks on tissue brain-derived neurotropic factor and tumor necrosis factor alpha in diabetic rats

**Fig. 5:**
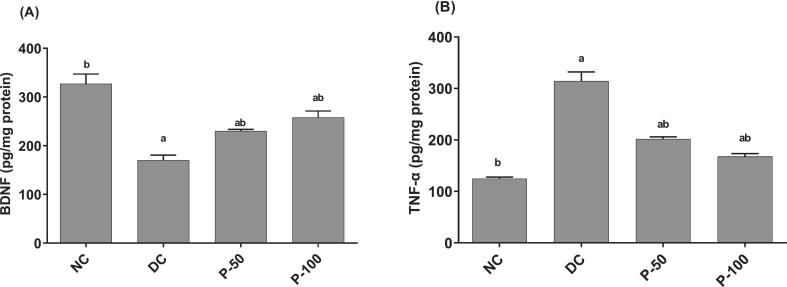
Effect of propolis on brain-derived neurotropic factor (BDNF) and tumor necrosis factor alpha (TNF-α) in brain tissue after 8 weeks in diabetic rats. ^a^Different in a significant way from the values of the negative control rats at *p* ≤ 0.05. ^b^Different in a significant way from the values of STZ/NA-DC rats at *p* ≤ 0.05.

Brain tissue content of brain-derived neurotropic factor (BDNF) reduced in a significant way in STZ/NA-DC rats as compared to negative control rats (Fig. 5). Compared to diabetic rats, propolis administration to diabetic rats (50 and 100 mg/kg), expressively increased serum and brain BDNF levels. Further, brain TNF-α levels substantially did increase in STZ/NA diabetic rats, with respect to negative control group. Propolis administration to diabetic rats (50 and 100 mg/kg), significantly decreased brain TNF-α levels as relative to the diabetic control rats.

### 3.8 Histopathological examination of pancreas and brain tissue after 8 weeks of propolis treatment

**Fig. 6:**
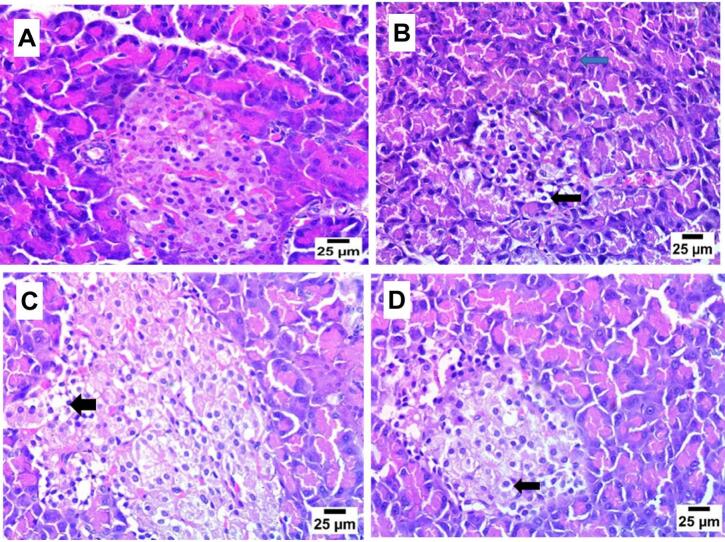
Effect of propolis on pancreas (H& E).

Fig. 6 illustrates that the pancreatic tissue of the NC group displayed normal histological structure of pancreatic acini and beta cells, whereas the pancreas tissue of the DC group exhibited necrobiotic changes in beta cells, including vacuolar degeneration and pyknotic nuclei with congestion of periacinal blood vessels and the presence of nuclear pyknosis in nuclei of pancreatic acini. Propolis-treated groups showed vacuolar degeneration in beta cells and pyknotic nuclei in some pancreatic acini.

**A.** Photomicrograph of NC category displaying normal histological state of pancreatic acini and beta cells. **B:** photomicrograph of DC group manifesting necrobiotic changes in beta cells including vacuolar degeneration and pyknotic nuclei (arrow) and presence of pyknosis in nuclei of pancreatic acini (blue arrow). **C:** photomicrograph of P-50 group showing necrobiotic changes in some beta cells including vacuolar degeneration and pyknotic nuclei (arrow). **D:** photomicrograph of P-100 group showing mild vacuolar degeneration in beta cells (arrow).

### 3.9 Histopathological examination of brain cerebral cortex and striatum after 8 weeks of propolis treatment

**Fig. 7:**
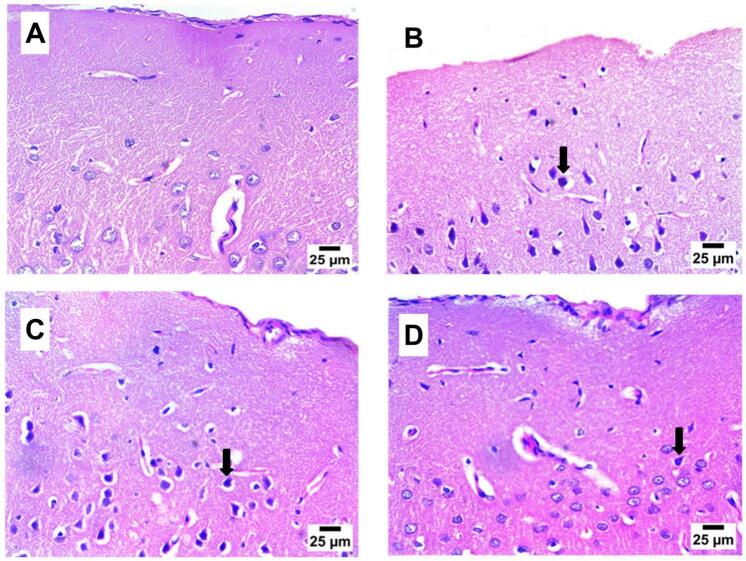
Effect of propolis on brain cerebral cortex after 8 weeks of propolis treatment (H& E).

Fig. 7 shows that the cerebral cortex of the NC group manifested typical histological structure, but the cerebral cortex of the DC group exhibited a large count of shrunk or emaciated and degenerated neurons with pyknotic nuclei. Propolis-treated groups demonstrated a high number of shrunken and degenerated neurons with pyknotic nuclei in the cerebral cortex of P-50 rats, as well as moderate nuclear pyknosis in the P-100 treated group.

**Fig. 8:**
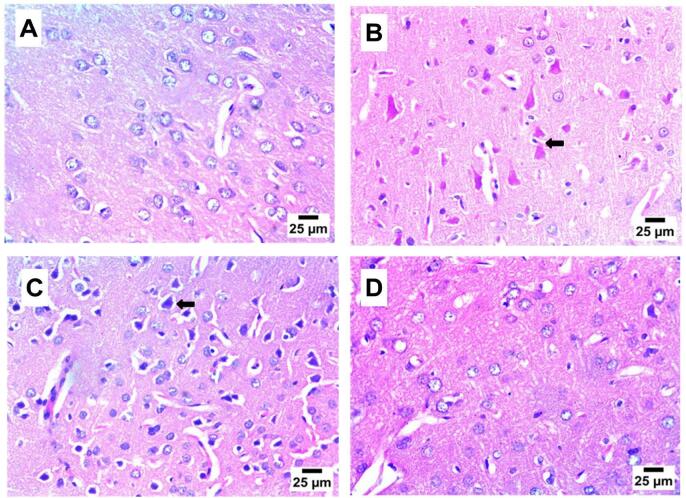
Effect of propolis on brain striatum after 8 weeks of propolis treatment (H& E).

Fig. 8 depicts that the striatum of the NC group exhibited normal histological structure, whereas the striatum of the DC group showed necrotic neurons with acidophilic cytoplasm and acidophilic nuclei. In the P-50 and P-100 treatment groups, there were moderate to low numbers of shrunken and degenerated neurons with pyknotic nuclei in the striatum.

**A:** photomicrograph of NC group displaying a typical structure, histologically, of cerebral cortex; **B:** photomicrograph of DC group showing high number of shrunken and degenerated neurons with pyknotic nuclei (arrow) in cerebral cortex; **C:** photomicrograph of P-50 group showing high number of shrunken and degenerated neurons with pyknotic nuclei (arrow) in cerebral cortex; **D:** photomicrograph of P-100 group showing mild nuclear pyknosis in cerebral cortex (arrow) (Hematoxylin and Eosin stain).

**A:** photomicrograph of NC group showing normal histological structure of striatum; **B:** photomicrograph of DC group showing necrotic neurons with acidophilic cytoplasm and acidophilic nuclei (arrow); **C:** photomicrograph of P-50 group showing moderate number of shrunken and degenerated neurons with pyknotic nuclei (arrow) in striatum; **D:** photomicrograph of P-100 group showing very low number of shrunken and degenerated neurons with pyknotic nuclei (arrow) in striatum (Hematoxylin and Eosin stain).

### 3.10 Immunohistochemical examination of GFAP in brain cerebral cortex and striatum after 8 weeks of propolis treatment

**Fig. 9:**
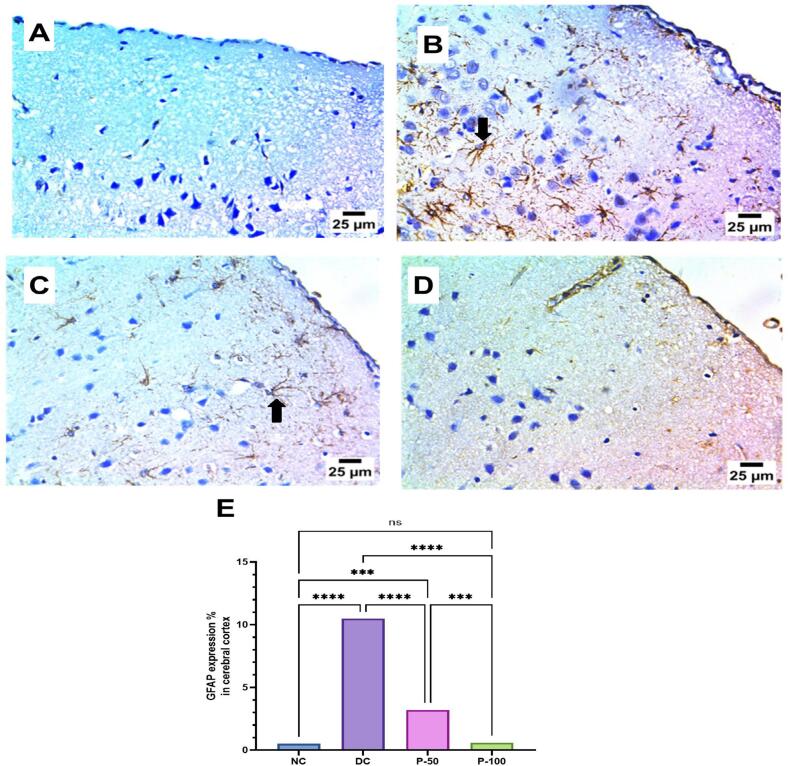
Effect of propolis on immunohistochemical examination of GFAP in brain cerebral cortex after 8 weeks of propolis treatment.

Immunohistochemical examination of GFAP, a marker for astrocyte activation, in the brain cerebral cortex or rats. In the cerebral cortex, the negative control group showed no GFAP expression, while DC group exhibited a severe positive reaction, indicating marked astrocyte activation. Propolis treatment at a lower dose (P-50) resulted in a mild positive reaction for GFAP, suggesting a reduction in astrocyte activation compared to the DC group. Notably, the higher dose of propolis (P-100) completely normalized GFAP expression, showing negative staining similar to the NC group (Fig. 9).

**Fig. 10:**
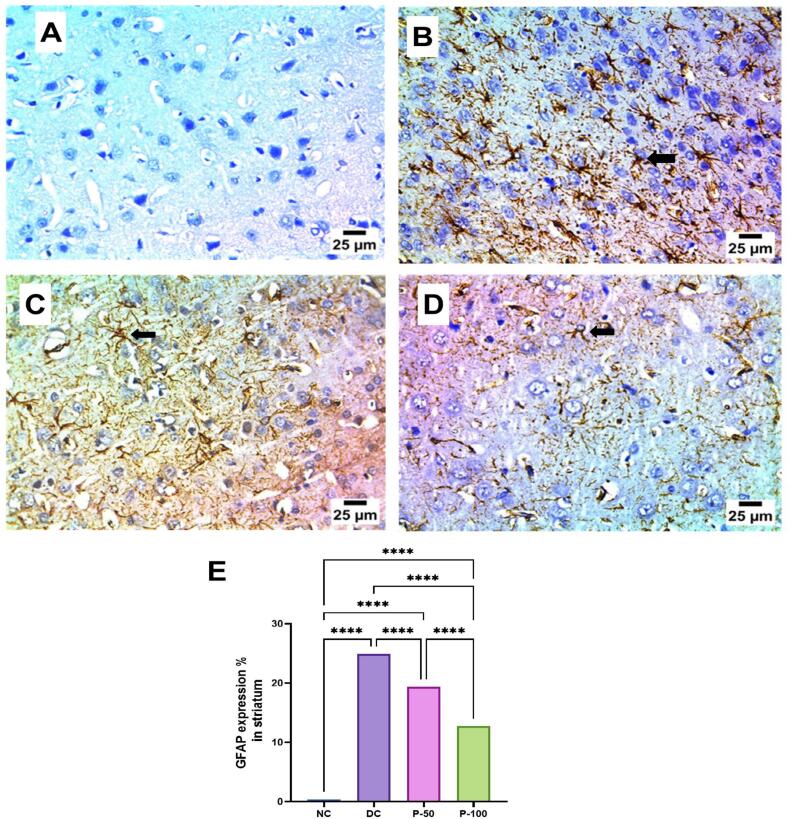
Effect of propolis on immunohistochemical examination of GFAP in brain striatum after 8 weeks of propolis treatment.

Immunohistochemical examination of GFAP in the brain striatum after 8 weeks of propolis treatment revealed notable changes in GFAP expression, indicative of astrocyte activation. The NC group showed negative GFAP expression in the striatum, while the DC group exhibited a severe positive reaction, reflecting significant astrocyte activation. Treatment with a lower dose of propolis (P-50) did not reduce GFAP expression, as the striatum still displayed a severe positive reaction similar to the DC group. However, the higher dose of propolis (P-100) resulted in a moderate positive reaction for GFAP, suggesting a partial reduction in astrocyte activation compared to the DC and P-50 groups (Fig. 10).

**A:** photomicrograph of NC group showing negative expression for GFAP in cerebral cortex; **B:** photomicrograph of DC group showing severe positive reaction for GFAP in cerebral cortex (arrow); **C:** photomicrograph of P-50 showing mild positive reaction for GFAP in cerebral cortex (arrow); **D:** photomicrograph of P-100 showing group negative expression for GFAP in cerebral cortex (IHC-Peroxidase-DAB). **E:** GFAP expression in the cerebral cortex was statistically significant at * at *P* ≤ 0.05, ** at *P* ≤ 0.01, *** at *P* ≤ 0.001, and **** at *P* ≤ 0.0001.

**A:** photomicrograph of NC group showing negative expression for GFAP in striatum; **B:** photomicrograph of DC group showing severe positive reaction for GFAP in striatum (arrow); **C:** photomicrograph of P-50 showing sever positive reaction for GFAP in striatum (arrow); **D:** photomicrograph of P-100 showing moderate positive reaction for GFAP in striatum (arrow) (IHC-Peroxidase-DAB); **E:** GFAP expression in the cerebral cortex was statistically significant at * at *P* ≤ 0.05, ** at *P* ≤ 0.01, *** at *P* ≤ 0.001, and **** at *P* ≤ 0.0001.

### 3.11 Effect of propolis treatment for 8 weeks on brain DNA fragmentation using COMET assay

**Fig. 11:**
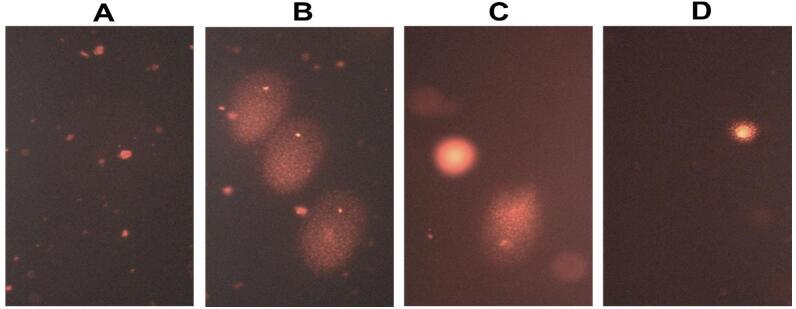
Images representing brain tissue analysis of a rat through comet assay.

Fig. 11 shows a photomicrograph (B) of damaged DNA in the diabetic rat group compared to the control group with intact undamaged DNA (A). Rat groups treated with propolis showed significant protection against DNA damage in brain tissue samples (C and D).

**Fig. 12:**
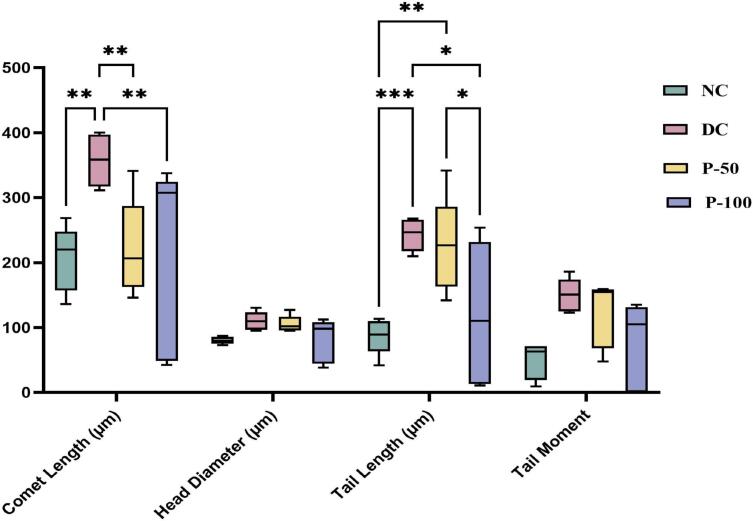
Effect of propolis on brain DNA damage using COMET assay after 8 weeks in diabetic rats.

The percentage of DNA in COMET length and tail length increased significantly (Fig. 12) in DC groups compared to NC groups. However, propolis administration reduced DNA damage to normal levels.

**(A)** a nucleoid revealing intact and an undamaged DNA (NC), **(B)** image showing damaged DNA (STZ/NA-DC), **(C)** moderate damaged DNA (STZ-P50), and **(D)** mild damaged DNA (STZ-P100).

The effect of propolis on the integrity of DNA was investigated through the use of the comet test. Data are shown as median with interquartile ranges (5 samples/group; 50 cells/sample) and minimum–maximum (I). Statistical significance relative to the NC group * at *P* ≤ 0.05, ** at *P* ≤ 0.01, *** at *P* ≤ 0.001, and **** at *P* ≤ 0.0001.

## 4 Discussion

Type 2 diabetes (T2DM) accounts for 90–95% f those with diabetes, accompanied by impaired insulin production and action. Evolving insulin resistance in type 2 diabetes, is primarily compensated by enhanced insulin secretion, with blood glucose levels remaining close to normal or slightly elevated.^51^ Patients with type 2 diabetes frequently experience obesity-related reduced insulin sensitivity as well as activated beta-cell compensatory mechanisms, such as excess basal insulin secretion and hyperproinsulinemia, as part of their metabolic profile.^47^

Streptozotocin (STZ)-nicotinamide (NA), one of the most frequently used rat models of T2DM, that is used to screening and testing potential antidiabetic agents. Low doses of STZ can induce partial damage to insulin-secreting beta cells rather than total cell death. Additionally, NA, a precursor to nicotinamide adenine dinucleotide (NAD), protects islet cells from damage caused by too much STZ by decreasing PARP-1 activity and boosting intracellular NAD. This ensures steady T2DM. Additionally, high-fat diet (HFD) provoke hyperlipidemia and markedly exacerbate insulin resistance in the STZ/NA rat. Based on this, a combination of STZ and NA causes pancreatic β-cells to undergo mild damage, which results in glucose intolerance. As a result, it is claimed that the NA-STZ model is more reflective of human T2DM and is useful for assessing the antidiabetic potential of pharmaceutical and natural agents.^26^, ^27^, ^5^, ^28^

Increased insulin secretion by pancreatic β-cells (hyperinsulinemia) initially compensates for beta cell malfunction and insulin resistance, which are early features of type 2 diabetes. Insulin resistance and decreased insulin secretion then work together to diminish insulin-mediated glucose uptake and utilization by skeletal muscle and inhibit insulin-mediated regulation of hepatic glucose production as these β-cells become “exhausted.” These metabolic disorders are made worse by ongoing endocrine control degradation, which also raises hyperglycemia.^29^, ^30^ As a result, increasing insulin sensitivity becomes a crucial goal for T2DM treatment.

Propolis at the two examined dose levels, significantly lowered the levels of FBG in comparison to STZ/NA-DC group after 4 and 8 weeks of treatment. Additionally, insulin levels of diabetic rats induced with propolis remarkably improved as relative to STZ/NA-DC after 4 and 8 weeks. Additionally, following 4 and 8 weeks of propolis administration (50 and 100 mg/kg), HOMA-IR was considerably stabilized in the propolis-treated rats compared to the STZ/NA-DC group. The current study's data are in line with earlier research showing that propolis can increase insulin sensitivity, regulate lipid metabolism, and regulate blood glucose in T2DM rats.^31^, ^30^ Further, propolis resulted in reduction in FBG and HbA1c levels and improved lipid profile in type 2 diabetic patients.^32^, ^33^

Dyslipidemia, a condition marked by changed plasma lipid profiles and an elevated risk of cardiovascular disease, is frequently linked to the onset of diabetes.^62^ Here in this investigation, serum lipid profile including cholesterol, TGs, and LDL of diabetic animals exhibited marked increase while HDL was declined when evaluated against the rats in the control group. On propolis treatment, serum lipid profile was substantially improved in dose dependent manner. The precise mechanisms by which propolis lowers LDL and raises HDL remain unknown. Yet, the ability of propolis to improve blood lipids may be in part linked to its antioxidant properties. It acts by modulating lipid metabolism, lowering lipid peroxidation (LPO) and scavenging free radicals.^38^

Ichi et al.^26^ found that propolis controlled lipid metabolism in a rat model of type 2 diabetes mellitus produced by alloxan. This could be explained by its association with key lipogenesis and lipolysis proteins such HMG-CoA reductase. Additionally, studies have shown that propolis's ethanolic extract and its subfractions are beneficial for increasing HDL by increasing the expression of the liver ATP-binding cassette transporters A1 and G1 (ABCA1 and ABCG1) protein, which is linked to peripheral tissue cholesterol efflux. Besides, Brazilian red propolis increased ApoA-1-mediated cholesterol efflux by macrophages, which is linked to ABCA1 through stimulation of transcription factors PPARϒ/LXR, involved in lipid metabolism.^27^

An imbalance between a biological system's capacity to readily detoxify reactive intermediates or repair the damage caused by reactive oxygen species (ROS) and the systemic generation of ROS is known as oxidative stress.^6^ By upsetting the regular redox state of cells, hyperglycemia can lead to oxidative stress. This can damage all cell components, including proteins, lipids, and DNA, by producing peroxides and free radicals.^56^ Furthermore, microvascular cerebral disorders like stroke, cerebral hemorrhage, and brain infarction can be brought on by elevated oxidative stress. The brain is more susceptible to oxidative damage than any other organ because it has a high concentration of readily oxidized unsaturated fatty acids and catecholamines and a low concentration of antioxidants, even though it uses 20% of the body's oxygen.^24^ Furthermore, an increase in MDA levels indicates cell membrane LPO and cell injury. likewise, SOD scavenges free radicals and regulates oxidative stress via many routes. SOD is capable of converting superoxide radicals into hydrogen peroxides (H2O2) and molecular oxygen, whereas catalase and peroxidases can convert H2O2 to water.^37^, ^40^

In the current study, rats' brain oxidative state significantly abruptly changed when they were given diabetes. When propolis was given to diabetic rats, the brain levels of MDA were much lower, but the brain levels of reduced glutathione and SOD activity were significantly higher than in the DC group, particularly in the higher dose group.

Propolis exhibits its antioxidant activity by inhibiting lipid peroxidation and scavenging ˙OH radicals. In order to combat free radicals, it also activates antioxidant enzymes such as catalase and superoxide dismutase. Propolis's flavonoid concentration, which can scavenge free radicals and hence prevent lipid peroxidation, is primarily responsible for its antioxidant effect.^41^, ^42^

Neuron-specific enolase (NSE) or specific neuron enolase (SNE) is an essential neuronal glycolytic enzyme found in brain gray matter neurons, and its synthesis increases following ischemia and axon injury. The physiological role of NSE is to regulate nerve cell growth and development as well as to participate in nerve cell energy metabolism.^43^, ^44^ Increased NSE levels may be suggestive of neuropathy and the extent of implicated nerve fiber injury, which are likely to be connected with changes in the synthesis and release of the enolase.^30^ Furthermore, it is already recognized that inflammation contributes to the development of diabetes and insulin resistance. Research indicates that the onset of insulin resistance and type 2 diabetes mellitus is preceded by elevated plasma levels of acute-phase proteins, specifically C-reactive protein (CRP).^39^

In the current investigation, diabetic rats had significantly higher blood and brain levels of NSE, CRP, and homocysteine than negative control rats. When propolis (50 and 100 mg/kg) was given to diabetic rats, the levels of NSE, CRP, and homocysteine in the brain and serum were significantly lower than in DC rats.

Meanwhile, homocysteine is a nonprotein amino acid and a methionine metabolite. Homocysteine can be transformed into methionine; however, dysregulated methionine metabolism leads to the increase of plasma homocysteine levels, known as hyperhomocysteinemia.^45^ Besides, homocysteine has been found to have a neurotoxic effect on ischemic brain cells, and in relation to cognitive dysfunction, it has equally been projected to be a risk factor. It is also an independent risk factor for dementia. In addition, oxidative stress is one of the key mechanisms of homocysteine-induced neurotoxicity.^48^, ^49^

According to the current study, propolis was able to reduce behavioral deterioration and cognitive dysfunction in mice's brains caused by the buildup of amyloid proteins and the plasma homocysteine-lowering impact.^36^ Furthermore, propolis mitigated the cellular damage parameters brought about by hyperhomocysteinemia, that led to ROS and LPO overproduction in human vascular endothelial cells (HUVECs).^14^

Propolis constituents including artepillin C, caffeic acid phenethyl ester, galangin, kaempferol, and quercetin were suggested to potentially form a novel class of dietary-derived antioxidant compounds with antiangiogenic qualities.^5^ Likewise, propolis has been shown to reduce serum levels of IL-6, CRP, and TNF-α, making it a potential supplemental therapy for certain chronic diseases.^20^

Brain-derived neurotrophic factor (BDNF) is a neurotrophin that has been associated in both systemic and peripheral inflammatory disorders, such as acute coronary syndrome and type 2 diabetes.^18^ Additionally, BDNF modulates neurotransmitters and promotes neural plasticity, both of which are essential for memory and learning.^11^ In addition, many investigations revealed that the antioxidant action of BDNF may be attributable to its ability to scavenge free radical ions or modulate antioxidant enzymes.^26^, ^27^

Results revealed that brain content of BDNF was significantly decreased in STZ/NA-DC rats as relative to the negative control rats. Propolis administration to diabetic rats (50 and 100 mg/kg), substantially increased brain BDNF level. In agreement with these findings, propolis treatment of rats with ischemia/perfusion (I/R) injury substantially elevated BDNF levels in their brains compared to I/R control rats.^1^

The proinflammatory cytokine known as tumor necrosis factor alpha (TNF-α) is generated by microglia during neuroinflammation and has both homeostatic and pathological roles in the CNS.^40^ It is an adipocytokine that has been linked to insulin resistance. TNF-α dysregulation has been linked to various human disorders, including type 2 diabetes.^50^

In STZ/NA diabetic rats, brain TNF-α levels were considerably higher than those of the negative control group. While, propolis significantly decreased brain TNF-α levels in compariosn to the diabetic rats in the control group. In this context, propolis significantly reduced NOS, NO, TNF-α, and caspase-3 expression in the brain stem, cerebral cortex, and cerebellum following kainic acid-induced excitotoxicity in rats. This demonstrated that propolis can protect neuronal damage and could be used as an adjuvant in brain and neurological illnesses and traumas.^49^

Free radical scavenging, inhibition of prostaglandin and cyclooxygenase synthesis and nitric oxide generation, and decrease in inflammatory cytokine release are the main processes behind propolis' anti-inflammatory action.^54^ Besides, flavonoids contents of propolis, are known for their antioxidant activity, which is attributable to their capacity to inhibit free radical production. Since reducing oxidative stress can reduce inflammation, propolis can also reduce inflammation through its anti-oxidative activity.^5^

The COMET test is an extremely sensitive method that may detect different DNA damage lesions that affect cell integrity and cause cell death. By exposing genetic material to an electric field, this method can identify single-strand and double-strand DNA breaks as well as the locations of partial base excision repair.^60^, ^61^

In line with the oxidative stress linked to diabetes, the data from the current investigation showed a significant increase in DNA damage in the diabetic control (DC) group when compared to the normal control (NC). Propolis treatment notably attenuated this damage, as evidenced by the statistically significant reduction in comet assay parameters (e.g., COMET length and tail length) compared to the DC group (*p* ≤ 0.05).

Consistent with the results of the present study, propolis dramatically decreased DNA fragmentation in the brain tissue of rats suffering from acute hepatic encephalopathy brought on by thioacetamide. The antioxidative capability of phenolic components and flavonoids contents of propolis may explain those findings by preventing the generation of free radicals and lowering oxidative DNA damage.^37^

Significant inflammatory histopathological changes along with positive glial fibrillary acidic protein (GFAP) is a key intermediate filament protein in astrocytes; its expression is examined to investigate astrocyte activation. It is an immunohistochemical indicator overexpressed in response to neuronal and astrocyte inflammatory injury.^22^ Many studies have indicated that STZ results in profuse cellular proliferation, neuroinflammation, and GFAP expression in immune-stained brain sections of rodents.

The current study revealed significant elevation in the GFAP immunohistochemical reactivity expressed as reaction area percent in brain tissue of diabetic rats. However, GFAP immunostaining in brain tissues of rats treated with propolis demonstrated significant decrease. Precisely, statistical analysis of GFAP expression across groups showed significant difference, suggesting that propolis, particularly at higher doses, may effectively mitigate astrocyte activation in the cerebral cortex, potentially offering neuroprotective benefits. Moreover, the obtained findings indicated that while the higher dose of propolis partially alleviated astrocyte activation in the striatum, it was less effective in the striatum compared to its impact on the cerebral cortex. Previous work by Agca et al.^3^ revealed that propolis ethanolic extract enhanced GFAP synthesis and fibrillogenesis, which was indicative of the proliferation of astrocytes prior to their inhibition.

On limitations, it is critical to note here that only two doses of propolis were evaluated, which limits full dose–response characterization. Future studies should include additional doses to define the therapeutic window. Further, the absence of a non-diabetic HFD control group limits differentiation between diet-induced and diabetes-specific effects. Therefore, the observed neuroprotective effects of propolis should be interpreted cautiously.

In conclusion, propolis has anti-neuroinflammatory properties that protect rats with NA/STZ-induced diabetes from neuroinflammation in their brain tissue. Significant increases in brain tissue contents of NSE, CRP, homocysteine, oxidative stress biomarkers, and TNF-α, along with notable inflammatory histopathological alterations and positive GFAP immunohistochemical staining, demonstrated that inducing diabetes in rats caused neuroinflammation. Propolis treatment has been shown to protect against neuroinflammation and improve inflammatory histopathological changes, as well as GFAP immunohistochemical staining in the cerebral cortex and striatum. As a result, propolis is believed to be a potential protective candidate for the management of neuroinflammation associated with diabetes.

## 5 Conclusion

In a nicotinamide-streptozotocin-induced diabetic rat model, the present findings well do demonstrate that propolis administration is associated with attenuation of diabetes-related cerebral oxidative stress, inflammatory responses, and also histopathological alterations. In a vital way, these effects suggest a potential neuroprotective role of propolis under experimental diabetic conditions but important to note, as this study was conducted in an animal model with a limited range of doses and biomarkers, the results should be interpreted cautiously. Ultimately, future studies incorporating comprehensive dose–response analyses, mechanistic investigations, and translational models are required to clarify the therapeutic relevance of propolis in diabetes-associated cerebral injury.

## 6 Ethics declaration

The animal study was approved by the Biomedical Research Ethics Committee, Umm AlQura University. The study was conducted in accordance with the local legislation and institutional requirements.

## 7 Funding declaration

This research received no external funding; however, experimental facilities and consumables were supported by the Department of Pharmacology and toxicology, Umm AlQura University.

## CRediT authorship contribution statement

**Ahmed M. Ashour:** Writing – review & editing, Writing – original draft, Visualization, Validation, Methodology, Investiga, tion.

## Declaration of competing interest

The author declares that they have no known competing financial interests or personal relationships that could have appeared to influence the work reported in this paper.

## Data Availability

The datasets presented in this article are not readily available due to privacy and/or ethical restrictions. The datasets that support the findings of this study are available from the corresponding author upon reasonable request.

## References

[1] Won S.J., Tang X.N., Suh S.W., Yenari M.A., Swanson R.A. (2011). Hyperglycemia promotes tissue plasminogen activator-induced hemorrhage by increasing superoxide production. Ann Neurol.

[2] Shukla V., Shakya A.K., Perez-Pinzon M.A., Dave K.R. (2017). Cerebral ischemic damage in diabetes: an inflammatory perspective. J Neuroinflammation.

[3] Gispen W.H., Biessels G.J. (2000). Cognition and synaptic plasticity in diabetes mellitus. Trends Neurosci.

[4] Wu Y., Ding Y., Tanaka Y., Zhang W. (2014). Risk factors contributing to type 2 diabetes and recent advances in the treatment and prevention. Int J Med Sci.

[5] Abdel-Rahman R.F., Ezzat S.M., Ogaly H.A. (2020). Ficus deltoidea extract down-regulates protein tyrosine phosphatase 1B expression in a rat model of type 2 diabetes mellitus: a new insight into its antidiabetic mechanism. J Nutr Sci.

[6] Dave K.R., Tamariz J., Desai K.M. (2011). Recurrent hypoglycemia exacerbates cerebral ischemic damage in streptozotocin-induced diabetic rats. Stroke.

[7] Ahangari Z., Naseri M., Vatandoost F. (2018). Propolis: chemical composition and its applications in endodontics. Iran Endod J.

[8] Gupta R.C. (2016).

[9] Pahlavani N., Malekahmadi M., Firouzi S. (2020). Molecular and cellular mechanisms of the effects of Propolis in inflammation, oxidative stress and glycemic control in chronic diseases. Nutr Metab Lond).

[10] Masiello P., Broca C., Gross R. (1998). Experimental NIDDM: development of a new model in adult rats administered streptozotocin and nicotinamide. Diabetes.

[11] Szkudelski T. (2012). Streptozotocin–nicotinamide-induced diabetes in the rat: Characteristics of the experimental model. Exp Biol Med.

[12] Yusufoglu H.S., Soliman G.A., Abdel-Rahman R.F. (2015). Antihyperglycemic and antihyperlipidemic effects of Ferula duranii in experimental type 2 diabetic rats. Int J Pharmacol.

[13] Ashour A.M. (2024). Propolis attenuates diabetes-induced testicular injury by protecting against DNA damage and suppressing cellular stress. Front Pharmacol.

[14] Trinder P. (1969). Determination of blood glucose using an oxidase-peroxidase system with a non-carcinogenic chromogen. J Clin Pathol.

[15] Anderson, E.A., Balon, T.W., Hoffman, R.P., Sinkey, C.A., Mark, A.L., 1992. Insulin increases sympathetic activity but not blood pressure in borderline hypertensive humans. Hypertens. (Dallas, Tex. 1979) 19, 621–627. 10.1161/01.hyp.19.6.621. 10.1161/01.hyp.19.6.621 1592458

[16] Matthews D.R., Hosker J.P., Rudenski A.S., Naylor B.A., Treacher D.F., Turner R.C. (1985). Homeostasis model assessment: insulin resistance and beta-cell function from fasting plasma glucose and insulin concentrations in man. Diabetologia.

[17] Saeedan A.S., Abdel-Rahman R.F., Soliman G.A., Ogaly H.A., Abdel-Kader M.S. (2023). Amentoflavone attenuates oxidative stress and neuroinflammation induced by cerebral ischemia/reperfusion in rats by targeting HMGB1-mediated TLR4/NF-κB signaling pathway. Saudi Pharm J SPJ off Publ Saudi Pharm Soc.

[18] Mansour D.F., Nada S.A., El-Denshary E.S., Omara E.A., Asaad G.F., Abdel-Rahman R.F. (2015). Milk whey proteins modulate endotoxemia-induced hepatotoxicity in rats. Int J Pharm Pharm Sci.

[19] Bradford M.M. (1976). A rapid and sensitive method for the quantitation of microgram quantities of protein utilizing the principle of protein-dye binding. Anal Biochem.

[20] Sun M., Zigman S. (1978). An improved spectrophotometric assay for superoxide dismutase based on epinephrine autoxidation. Anal Biochem.

[21] Ellman G.L. (1959). Tissue sulfhydryl groups. Arch Biochem Biophys.

[22] Jain S.K., Levine S.N., Duett J., Hollier B. (1990). Elevated lipid peroxidation levels in red blood cells of streptozotocin-treated diabetic rats. Metabolism.

[23] John D. Bancroft and Marilyn Gamble, 2007. Theory and Practice of Histological Techniques, 6th ed.

[24] Szkudelska K., Nogowski L., Szkudelski T. (2014). Adipocyte dysfunction in rats with streptozotocin-nicotinamide-induced diabetes. Int J Exp Pathol.

[25] Skovsø S. (2014). Modeling type 2 diabetes in rats using high fat diet and streptozotocin. J Diabetes Investig.

[26] Wu C.-C., Hung C.-N., Shin Y.-C., Wang C.-J., Huang H.-P. (2016). Myrciaria cauliflora extracts attenuate diabetic nephropathy involving the Ras signaling pathway in streptozotocin/nicotinamide mice on a high fat diet. J Food Drug Anal.

[27] Wu C.-L., Chen S.-D., Yin J.-H., Hwang C.-S., Yang D.-I. (2016). Nuclear factor-kappaB-dependent Sestrin2 induction mediates the antioxidant effects of BDNF against mitochondrial inhibition in rat cortical neurons. Mol Neurobiol.

[28] Yan L.-J. (2022). The Nicotinamide/Streptozotocin Rodent Model of Type 2 Diabetes: Renal Pathophysiology and Redox Imbalance Features. Biomolecules.

[29] Cerf M.E. (2013). Beta cell dysfunction and insulin resistance. Front Endocrinol (Lausanne).

[30] Li Y., Chen M., Xuan H., Hu F. (2012). Effects of encapsulated propolis on blood glycemic control, lipid metabolism, and insulin resistance in type 2 diabetes mellitus rats. Evid Based Complement Alternat Med.

[31] Fuliang H.U., Hepburn H.R., Xuan H., Chen M., Daya S., Radloff S.E. (2005). Effects of propolis on blood glucose, blood lipid and free radicals in rats with diabetes mellitus. Pharmacol Res.

[32] Zakerkish M., Jenabi M., Zaeemzadeh N., Hemmati A.A., Neisi N. (2019). The effect of iranian propolis on glucose metabolism, lipid profile, insulin resistance, renal function and inflammatory biomarkers in patients with type 2 diabetes mellitus: a randomized double-blind clinical trial. Sci Rep.

[33] Samadi N., Mozaffari-Khosravi H., Rahmanian M., Askarishahi M. (2017). Effects of bee propolis supplementation on glycemic control, lipid profile and insulin resistance indices in patients with type 2 diabetes: a randomized, double-blind clinical trial. J Integr Med.

[34] Mujica V., Orrego R., Pérez J. (2017). The role of propolis in oxidative stress and lipid metabolism: A randomized controlled trial. Evid Based Complement Alternat Med.

[35] Ichi I., Hori H., Takashima Y. (2009). The beneficial effect of propolis on fat accumulation and lipid metabolism in rats fed a high-fat diet. J Food Sci.

[36] Iio A., Ohguchi K., Maruyama H. (2012). Ethanolic extracts of Brazilian red propolis increase ABCA1 expression and promote cholesterol efflux from THP-1 macrophages. Phytomedicine.

[37] Alsharif I.A., Fayed H.M., Abdel-Rahman R.F., Abd-Elsalam R.M., Ogaly H.A. (2022). Miconazole mitigates acetic acid-induced experimental colitis in rats: insight into inflammation, oxidative stress and Keap1/Nrf-2 signaling crosstalk. Biology (Basel).

[38] Wolff S.P. (1993). Diabetes mellitus and free radicals. Free radicals, transition metals and oxidative stress in the aetiology of diabetes mellitus and complications. Br Med Bull.

[39] Hong J.-H., Kim M.-J., Park M.-R. (2004). Effects of vitamin E on oxidative stress and membrane fluidity in brain of streptozotocin-induced diabetic rats. Clin Chim Acta.

[40] Eldesoky A.H., Abdel-Rahman R.F., Ahmed O.K. (2018). Antioxidant and hepatoprotective potential of plantago major growing in egypt and its major phenylethanoid glycoside, acteoside. J Food Biochem.

[41] Attia A.A., ElMazoudy R.H., El-Shenawy N.S. (2012). Antioxidant role of propolis extract against oxidative damage of testicular tissue induced by insecticide chlorpyrifos in rats. Pestic Biochem Physiol.

[42] Ramadan A., Soliman G., Mahmoud S.S., Nofal S.M., Abdel-Rahman R.F. (2015). Hepatoprotective and hepatotheraputic effects of propolis against d-galactosamine/lipopolysaccharide-induced liver damage in rats. Int J Pharm Pharm Sci.

[43] Humaloja J., Ashton N.J., Skrifvars M.B. (2022). Brain injury biomarkers for predicting outcome after cardiac arrest. Crit Care.

[44] Liu F., Li H., Hong X., Liu Y., Yu Z. (2024). Research progress of neuron-specific enolase in cognitive disorder: a mini review. Front Hum Neurosci.

[45] Li J., Zhang H., Xie M., Yan L., Chen J., Wang H. (2013). NSE, a potential biomarker, is closely connected to diabetic peripheral neuropathy. Diabetes Care.

[46] Nesto R. (2004). C-reactive protein, its role in inflammation, Type 2 diabetes and cardiovascular disease, and the effects of insulin-sensitizing treatment with thiazolidinediones. Diabet Med.

[47] Sen U., Tyagi S.C. (2010). Homocysteine and hypertension in diabetes: does PPARγ have a regulatory Role?. PPAR Res.

[48] Miyazaki Y., Sugimoto Y., Fujita A., Kanouchi H. (2015). Ethanol extract of Brazilian propolis ameliorates cognitive dysfunction and suppressed protein aggregations caused by hyperhomocysteinemia. Biosci Biotech Bioch.

[49] Wang L., Niu H., Zhang J. (2020). Homocysteine induces mitochondrial dysfunction and oxidative stress in myocardial ischemia/reperfusion injury through stimulating ROS production and the ERK1/2 signaling pathway. Exp Ther Med.

[50] Darendelioglu E., Aykutoglu G., Tartik M., Baydas G. (2016). Turkish propolis protects human endothelial cells in vitro from homocysteine-induced apoptosis. Acta Histochem.

[51] Ahn M.-R., Kunimasa K., Kumazawa S. (2009). Correlation between antiangiogenic activity and antioxidant activity of various components from propolis. Mol Nutr Food Res.

[52] Gholami A., Dinarvand N., Hariri M. (2024). Propolis supplementation can reduce serum level of interleukin-6, C-reactive protein, and tumor necrosis factor-α: an updated systematic review and dose-response meta-analysis on randomized clinical trials. J Heal Popul Nutr.

[53] Eyileten C., Kaplon-Cieslicka A., Mirowska-Guzel D., Malek L., Postula M. (2017). Antidiabetic effect of brain-derived neurotrophic factor and its association with inflammation in type 2 diabetes mellitus. J Diabetes Res.

[54] Bathina S., Das U.N. (2015). Brain-derived neurotrophic factor and its clinical Implications. Med Sci Arch.

[55] Abdel-Rahman R.F., Alqasoumi S.I., Ogaly H.A., Abd-Elsalam R.M., El-Banna H.A., Soliman G.A. (2020). Propolis ameliorates cerebral injury in focal cerebral ischemia/reperfusion (I/R) rat model via upregulation of TGF-β1. Saudi Pharm J.

[56] Olmos G., Lladó J. (2014). Tumor necrosis factor alpha: a link between neuroinflammation and excitotoxicity. Mediators Inflamm.

[57] Swaroop J.J., Rajarajeswari D., Naidu J.N. (2012). Association of TNF-α with insulin resistance in type 2 diabetes mellitus. Indian J Med Res.

[58] Swamy M., Suhaili D., Sirajudeen K.N.S., Mustapha Z., Govindasamy C. (2014). Propolis ameliorates tumor nerosis factor-α, nitric oxide levels, caspase-3 and nitric oxide synthase activities in kainic acid mediated excitotoxicity in rat brain. African J Tradit Complement Altern Med AJTCAM.

[59] Trusheva B., Todorov I., Ninova M., Najdenski H., Daneshmand A., Bankova V. (2010). Antibacterial mono- and sesquiterpene esters of benzoic acids from Iranian propolis. Chem Cent J.

[60] Mostafa R.E., Salama A.A.A., Abdel-Rahman R.F., Ogaly H.A. (2017). Hepato-and neuro-protective influences of biopropolis on thioacetamide-induced acute hepatic encephalopathy in rats. Can J Physiol Pharmacol.

[61] Bankoglu E.E., Schuele C., Stopper H. (2021). Cell survival after DNA damage in the comet assay. Arch Toxicol.

[62] Guo L., Li Y., Li W. (2022). Shikonin ameliorates oxidative stress and neuroinflammation via the Akt/ERK/JNK/NF-κB signalling pathways in a model of Parkinson’s disease. Clin Exp Pharmacol Physiol.

[63] Agca C.A., Tykhomyrov A.A., Baydas G., Nedzvetsky V.S. (2017). Effects of a propolis extract on the viability of and levels of cytoskeletal and regulatory proteins in rat brain astrocytes: an in vitro study. Neurophysiology.

